# Structural Stability of NaCl and KCl Cleavage Surfaces in the BMIM-PF_6_ Ionic Liquid

**DOI:** 10.1021/acs.langmuir.5c00163

**Published:** 2025-05-30

**Authors:** Ebru Cihan, Natalia Janiszewska, Kamil Awsiuk, Qingwei Gao, Rong An, Ronen Berkovich, Enrico Gnecco

**Affiliations:** † Institute for Materials Science and Max Bergmann Center for Biomaterials, TU Dresden, Dresden 01069, Germany; ‡ Marian Smoluchowski Institute of Physics, Faculty of Physics, Astronomy and Applied Computer Science, 37799 Jagiellonian University, Krakow 30348, Poland; § National Synchrotron Radiation Centre SOLARIS, Jagiellonian University, Czerwone Maki 98, Krakow PL-30392, Poland; ∥ College of Environmental and Chemical Engineering, Shanghai Key Laboratory of Materials Protection and Advanced Materials in Electric Power, 12596 Shanghai University of Electric Power, Shanghai 200090, China; ⊥ School of Materials Science and Engineering/Herbert Gleiter Institute of Nanoscience, 12436 Nanjing University of Science and Technology, Nanjing 210094, China; # Shandong Laboratory of Advanced Materials and Green Manufacturing at Yantai, Yantai 264006, P. R. China; ¶ Department of Chemical Engineering, 26732 Ben-Gurion University of the Negev, Beer-Sheva 8410501, Israel; ∇ Ilze Katz Institute for Nanoscience and Technology, Ben-Gurion University of the Negev, Beer-Sheva 8410501, Israel

## Abstract

We have investigated the evolution of freshly cleaved NaCl(100) and KCl(100) surfaces exposed to the ionic liquid (IL) 1-butyl-3-methylimidazolium hexafluorophosphate (BMIM-PF_6_) and repeatedly scraped using atomic force microscopy (AFM). The response of the two surfaces to the IL is completely different. On NaCl, the cleavage step edges are slightly eroded, and the surface is progressively smoothed by the AFM tip. These changes are accompanied by a continuous increase in the friction force. On KCl, a dramatic dissolution of the surface is observed immediately after bringing it into contact with the IL. The surface is then smeared along the fast scan direction in the area scratched by the tip and even beyond. An increase in the friction force is also observed but only in the beginning of the surface modification process. Crystallites (∼100–200 nm in size) are observed all over the unscratched areas of KCl but not of NaCl. This result is supported by molecular dynamics simulations and Raman spectroscopy, which indicate a much stronger interaction of the IL with the KCl surface and, respectively, the formation of a BMIM-PF_6_ solid phase on it. The analysis performed on the model systems presented here could be extended to other ionic crystal surfaces in contact with ILs, the possible degradation of which must be evaluated in view of their use in catalysis and energy storage applications.

## Graphical abstract

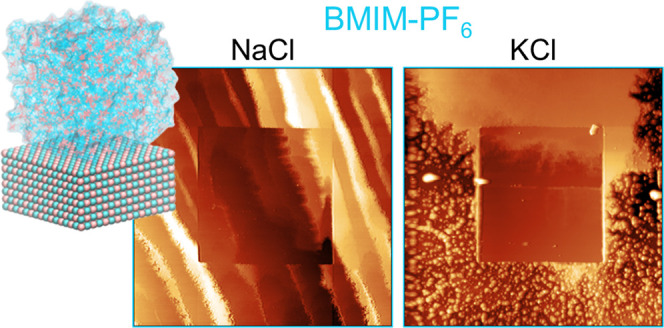

## 1 Introduction

Ionic liquids (ILs) have garnered significant attention in recent years as potential replacements for conventional organic solvents owing to their unique and advantageous properties. These compounds exhibit remarkably low volatility, ensuring minimal evaporation and thus providing enhanced stability in prolonged processes. Additionally, their nonflammable nature contributes to improved safety across a wide range of applications. ILs also demonstrate exceptional thermal stability, maintaining their integrity at elevated temperatures without decomposition, which makes them particularly suitable for high-temperature operations. These distinctive characteristics collectively contribute to ILs’ unique structural phase in the bulk. Interestingly, this bulk phase can undergo substantial modifications when in contact with solid surfaces, a phenomenon that has implications for its behavior in various systems and applications. The combination of these properties positions ILs as versatile and promising candidates for numerous industrial and scientific applications where conventional solvents may fall short.^1^

The interaction of ILs with a solid surface can lead to different structural arrangements at the interface^2,3^ and exhibit fascinating behaviors, particularly when ILs form ordered or solid-like layers adjacent to crystalline substrates.^4–8^ The ordered layers can affect the physical properties of the IL, such as viscosity and conductivity, and can also affect the overall performance of the IL in various surface chemistry applications. Uncovering the interfacial structural organization of ILs is also important for optimizing their use in technologies such as batteries^9^ and catalysis.^10^ In this context, investigating the response of crystal surfaces experiencing strong electrostatic interactions, such as alkali halides, can provide insights into peculiar arrangements of the IL molecules at the interface^3^ also in view of the aforementioned applications. Nevertheless, experimental studies combining ILs with alkali halide surfaces at the nanoscale are scarce,^2,3,7,8^ and important issues such as the stability of these surfaces in ILs remain unexplored. By stability, we mean the resistance of the surface to morphological changes caused by the contact with the IL or by external perturbations such as mechanical abrasion, also in the presence of the IL. Both issues can be investigated *simultaneously* using atomic force microscopy (AFM), as demonstrated here in the case of KCl and NaCl cleavage surfaces exposed to the IL 1-butyl-3-methylimidazolium hexafluorophosphate (BMIM-PF_6_) for 6–7 h at relatively low normal loads (*F*_N_ < 10 nN). In addition, we have also recurred to Raman spectroscopy and molecular dynamics (MD) simulations to interpret the different responses of the two surfaces scratched or left unperturbed in the IL.

The structural properties of NaCl and KCl crystals, along with the physicochemical characteristics of BMIM-PF_6_, play a critical role in understanding their interfacial behavior. Both NaCl and KCl crystallize in the halite structure with surface orientations and energies that vary based on growth conditions. For NaCl, the (100) surface is the most thermodynamically stable, featuring alternating Na^+^ and Cl^–^ ions in a charge-neutral configuration, with a relatively low surface energy of 160 erg/cm^2^. In contrast, the polar (111) faces exhibit significantly higher surface energies (390–405 erg/cm^2^) and are rarely observed due to electrostatic instability under equilibrium conditions.^11^ Similarly, KCl generally forms (100) surfaces.

The IL BMIM-PF_6_, composed of a flexible imidazolium cation and a hexafluorophosphate anion, is characterized by hydrophobicity, thermal stability, and solid-state polymorphism.^12^ Its low water solubility (<12.5%) and high viscosity (0.207 Pa·s at 25 °C),^13^ along with hydrolytic instability of the PF_6_^–^ anion under acidic conditions,^14^ influence both its performance and compatibility in interfacial environments. At salt–liquid interfaces, key factors include electrostatic interactions between IL species and the surface ions of the salts, hydrophobic effects that limit water-mediated dissolution, and conformational adaptability of the [BMIM]^+^ cation, which enables dynamic restructuring on the crystal surface. These properties are particularly relevant for applications in corrosion inhibition, electrochemical devices, and nanomaterial synthesis.

## 2 Materials and Methods

### 2.1 AFM Characterization

NaCl and KCl single crystals (Ted Pella, USA) were cleaved manually and imaged with a Multimode AFM equipped with a Nanoscope IIIA Control Station (Bruker, USA) in ambient conditions (*T* = 23 °C, 45% relative humidity) in contact mode and ultralow normal forces (*F*_N_ < 10 nN). SNL probes from Bruker, USA, with a nominal resonance frequency of 18 kHz, a nominal spring constant of 0.06 N/m, and a nominal radius of curvature of 2 nm were used. Afterward, drops of the IL 1-butyl-3-methylimidazolium hexafluorophosphate (BMIM-PF_6_, Sigma-Aldrich, Germany) were poured on the samples and also on the AFM cantilevers to prevent the sudden formation of capillary bridges between the IL and the otherwise dry tip when the last one approached the sample. The resulting surfaces were imaged again in the IL for 6–7 h. The lateral forces accompanying the topography images were calibrated based on the equation introduced by Noy et al. for *V*-shaped probes.^15^ Friction maps were obtained by subtracting the lateral force signals recorded during forward and backward scanning.

### 2.2 Raman Spectroscopy

To investigate possible chemical changes on the NaCl and KCl surfaces exposed to BMIM-PF_6_, Raman spectroscopy (Alpha 300R, WITec, Ulm, Germany) was used. For this purpose, the IL was blown off the crystal surfaces by using a nitrogen gun. The analysis was performed on 10 × 10 μm^2^ areas scanned with a 532 nm wavelength laser (20 mW power and 50× objective) with 20 points per line and 2 s integration time.

### 2.3 Molecular Dynamics Simulations

MD simulations were performed in a box with a volume of 5.5 × 5.6 × 8.5 nm^3^ (Figure 1). For both NaCl and KCl surfaces, we constructed the crystal using approximately eight atomic layers. 300 [BMIM]^+^ and [PF_6_]^−^ pairs described by the OPLS-AA force field^16,17^ were put on each surface. The 12-6 Lennard-Jones (LJ) potential combining a Coulombic potential was used to describe the intermolecular interactions:

1 $$U\left(r_{ij}\right)=4\varepsilon_{ij}\left[{\left(\frac{\sigma_{ij}}{r_{ij}}\right)}^{12}-{\left(\frac{\sigma_{ij}}{r_{ij}}\right)}^{6}\right]+\frac{q_{i}q_{j}}{r_{ij}}$$

**Figure 1:**
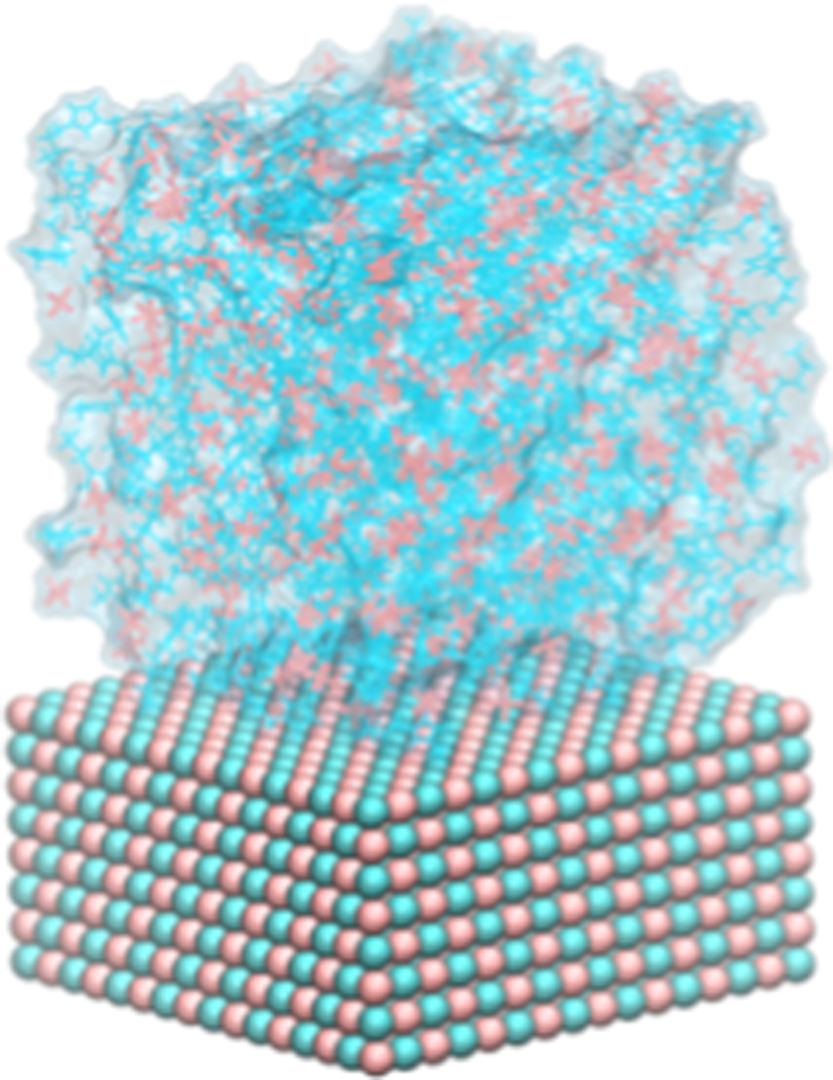
Molecular configuration of the [BMIM]^+^ cations (blue) and [PF_6_]^−^ anions (red) on a KCl(100) surface.

The Lorentz–Berthelot mixing rules were chosen to calculate the LJ parameters of the cross-interactions. Periodic boundary conditions were applied to all three dimensions. The particle mesh Ewald method^18^ was used to calculate the long-range electrostatic interaction with a cutoff for a real space of 1.2 nm. The short-range van der Waals cutoff was set to be 1.2 nm. All of the cases, after energy minimization, were first equilibrated in a 5 ns *NPT* ensemble and a 15 ns *NVT* ensemble, respectively, and every production run was performed for 5 ns in the *NVT* ensemble with a 2 fs time step and saved every 0.2 ps. The temperature in the system was maintained at 298.15 K, being controlled by a Nosé–Hoover thermostat.^19^ The Berendsen pressure coupling regulated the system at 1 bar. All simulations were performed using the MD package GROMACS 2024.1.^20^

## 3 Results and Discussion

### 3.1 Morphological Changes of Scratched and Unscratched NaCl and KCl Surfaces

Figure 2a–d shows the AFM topography images of the freshly cleaved NaCl and KCl surfaces measured in ambient conditions and, respectively, after a half hour of contact with BMIM-PF_6_ (on different areas). While the NaCl surface did not show noticeable changes, KCl appears considerably etched, with the original step edges disappeared and depressions up to ∼35 nm deep formed on the surface. The same regions were imaged again after 6 and 7 h, respectively (Figure 2c,d), after scratching their central parts (within the dashed blue lines) repeatedly (40 times for NaCl and 46 times for KCl) with the AFM tip. As a result, the step edges of NaCl appear displaced to the right. They also became jagged, and not only in the scratched area. The modification of the KCl surface caused by scratching was different and more substantial, as detailed below. Quite noticeably, out of the scratched area on KCl crystallites of about 100 nm in size were formed, which was not the case for NaCl.

**Figure 2:**
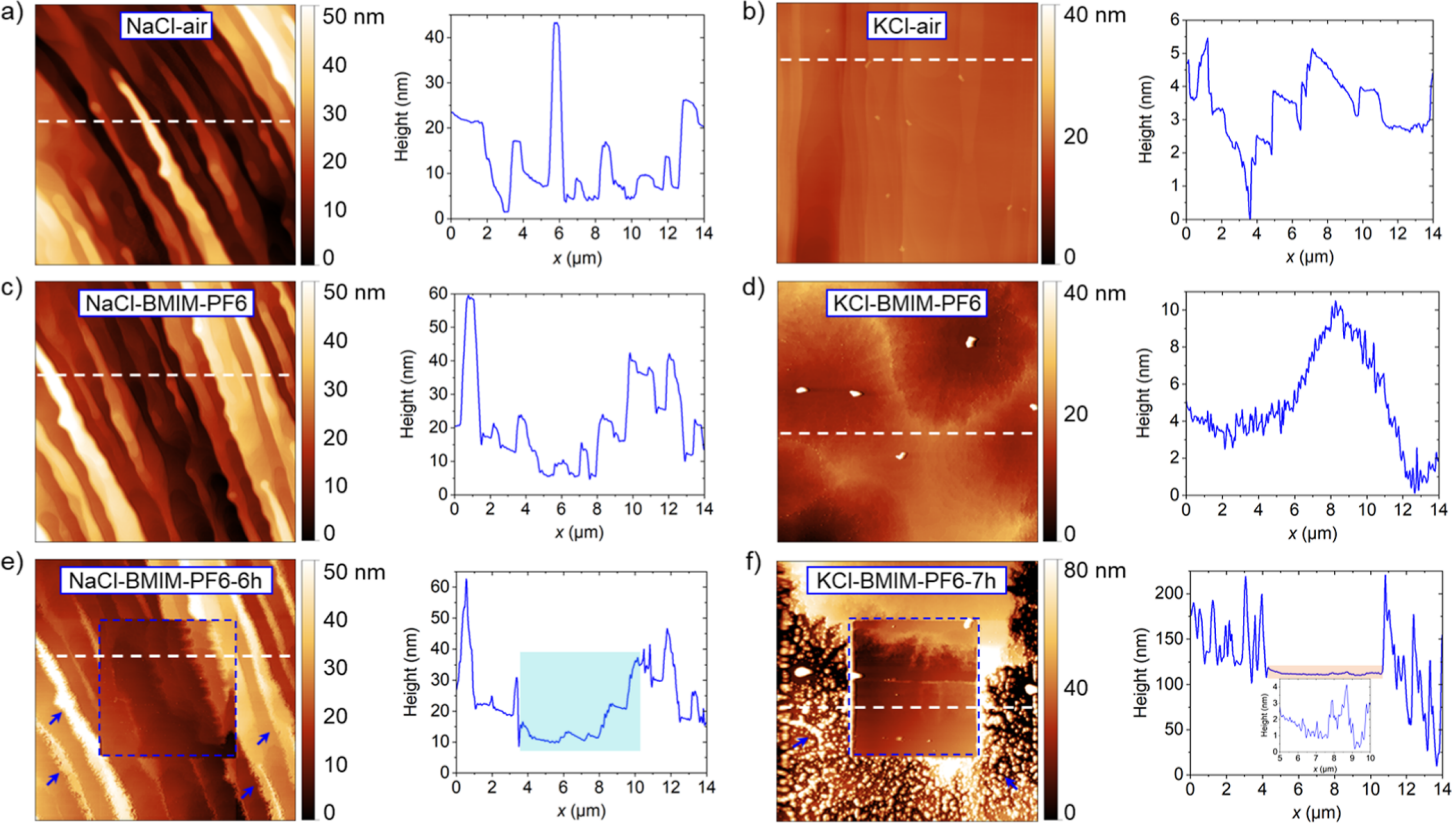
AFM topography images of NaCl and KCl surfaces measured (a,b) in ambient conditions and (c,d) in BMIM-PF_6_ IL. (e,f) AFM topography images of the same regions of NaCl and KCl left for 6 h or, respectively, 7 h in the IL. The blue dashed squares indicate the area continuously scratched by the AFM tip. The small (blue) arrows indicate the jagged-shaped structures formed on NaCl and, respectively, crystallites formed on KCl outside the scratched area. Frame sizes: 14 × 14 μm^2^. Normal force: *F*_N_ < 10 nN.

Two sequences of images documenting the evolution of the NaCl and KCl surfaces while scratched by the AFM tip are shown in Figure 3a (see also Supporting Information Videos S1 and S2). The NaCl surface is visibly smoothed in the sequence with an average decrease rate of the RMS roughness of 0.52 nm/h (Figure 4a). Similar to the pioneer results presented by Dickinson on a different ionic crystal (brushite) scraped in a supersaturated solution,^21^ atomic layer regrowth is triggered at the step edges, and this process tends to produce atomically smooth surfaces by “filling” rather than “polishing” the surface. The filling process also results in considerable displacement of the step edges along the fast scan direction. From Figure 2e, we can see that the total displacement is 1 μm after 6 h. In contrast, the KCl is progressively “smeared” upward by the tip when scratched (Figure 3b). A series of ripples formed in the bottom-top direction marks the transition of the moving layer. A second layer is formed after 4 h. It is also smeared up, while the first layer is merging into it. The surface roughness increases thorough the whole process, as quantified in Figure 4b.

**Figure 3:**
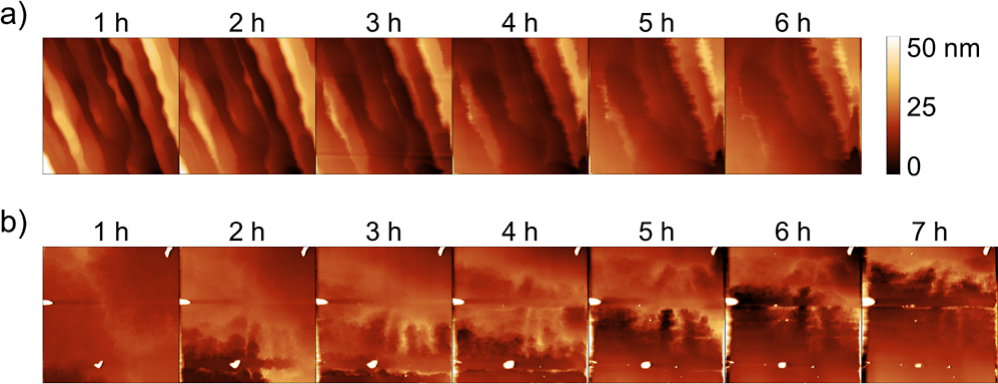
AFM topography images showing the evolution of the (a) NaCl and (b) KCl surface scratched in BMIM-PF_6_ over 6 or 7 h. Frame sizes: 7 × 7 μm^2^.

**Figure 4:**
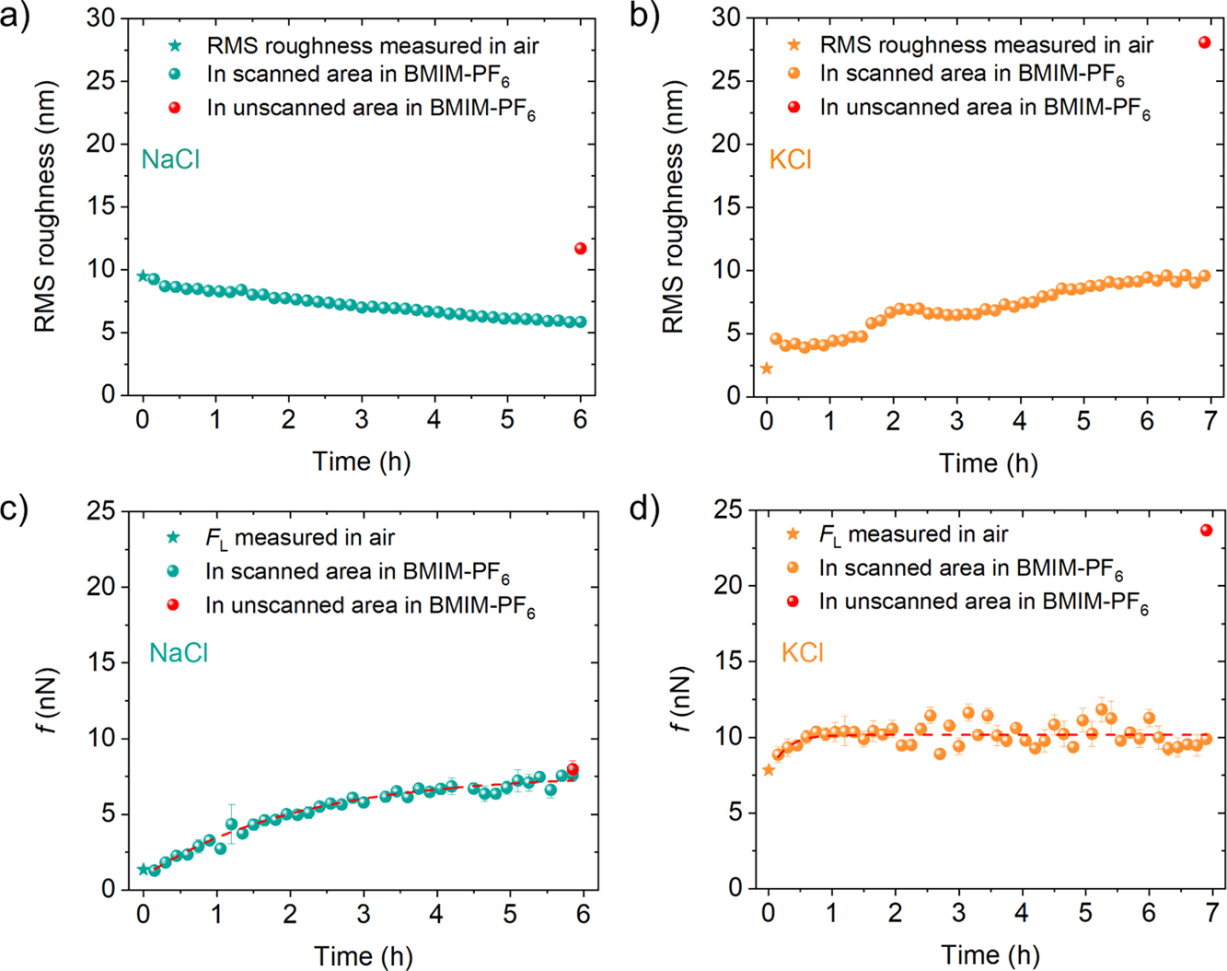
(a,b) Evolution of the RMS roughness of NaCl and KCl surfaces in BMIM-PF_6_ as they are repeatedly scratched by the AFM probe. (c,d) Corresponding variation of the average friction forces. The red dots show values of the RMS roughness and friction force measured on unscratched areas of the two surfaces.

### 3.2 Nanotribological Response

It is also interesting to see how the average friction force, *f*, evolves in the two sequences. The values of *f* are estimated from the friction force maps corresponding to the topographies in Figure 3 provided in the Supporting Information (Figures S1 and S2). As shown in Figure 4c,d, the friction *f* increases, in both cases, according to the empirical relation^22^

2 $$f=f_{0}e^{-t/\tau }+f_{1}\left(1-e^{-t/\tau }\right)$$

where *t* is the time elapsed from the beginning of the repeated scratching process. On NaCl, the initial and asymptotic values of the friction are *f*_0_ = 0.9 ± 0.08 nN and *f*_1_ = 7.65 ± 0.13 nN, and the characteristic time of the process τ = 2.1 ± 0.12 h. On KCl, *f* initially increases and then stabilizes within 1 h, when the process of ripple formation and displacement begins (Figure 4d). The red dot in Figure 4c shows that at the end of the modification process, the friction is the same on the unscratched and scratched parts of NaCl. On the contrary, it is two times larger on the scratched area of KCl (red dot in Figure 4d) as compared to the unscratched KCl. This difference is related to the formation of crystallites and is further discussed in Section 3.3.

The empirical relation eq 2 was proposed by Gnecco et al. to describe the behavior of KBr (100) repeatedly scratched (along the same line) in UHV.^22^ Assuming that the friction is proportional to the contact area *A*_con_ between the tip and the evolving surface, eq 2 means that the difference between the equilibrium value and the actual value of *A*_con_ relaxes exponentially with characteristic time τ. For KBr in UHV, the change in *A*_con_ was attributed to the formation of the surface ripples, the repetition distance of which is comparable with the tip size.^22^ In BMIM-PF_6_, we have already noticed (Figure 3b) that surface ripples are also formed on KCl, but rather than remaining stable on the surface, they are progressively displaced upward as the scanning is repeated. And the ripple formation begins after 1 h, when the friction force simply starts to fluctuate around the asymptotic value *f*_1_ = 10.16 ± 0.1 nN (Figure 4d), initiating at *f*_0_ = 7.52 ± 2.68 nN with a characteristic time of τ = 0.25 ± 0.22 h. The temporal friction evolution on KCl is substantially faster than that observed for the NaCl surface, which is still growing after 6 h (Figure 4c). Here, no ripples are visible, but a detailed observation of Figure 2e shows that the cleavage step edges of NaCl are not only eroded but also become jagged. This can in turn increase the contact area between the tip and surface (and of the friction force), while the RMS roughness of NaCl decreases. Since the final value of the friction is the same on the unscratched area (red dot in Figure 4c), we conclude that the “jagging” process is caused by the contact between NaCl and IL interaction, with no influence of interfacial shear stress caused by the sliding tip.

### 3.3 BMIM-PF_6_ Crystal Adsorbate Formation on KCl

On KCl, the formation of adsorbate structures in the areas that were not scratched by the AFM tip is suggested not only by the increase in the friction force by a factor 2 (Figure 4d) but also by the much more remarkable increase of the surface roughness by 1 order of magnitude, as seen from the comparison of the red dot in Figure 4b with the initial value of the RMS roughness plotted in the same figure. To shed light on the structure of the adsorbates, we have prepared a second KCl surface with larger mismatch with respect to the ⟨100⟩ direction and also put and kept it in contact with BMIM-PF_6_. Figure 5a shows a representative image of the surface after 6 h of contact. The material contrast observed in the corresponding friction map (Figure 5b) allows us to distinguish adsorbates along two major cleavage steps and perpendicular to them. Other snapshots at different locations of the evolving surface (not scratched by the tip) are shown in the Supporting Information (Figures S3 and S4), together with the KCl surface freshly cleaved and not yet covered by BMIM-PF_6_.

**Figure 5:**
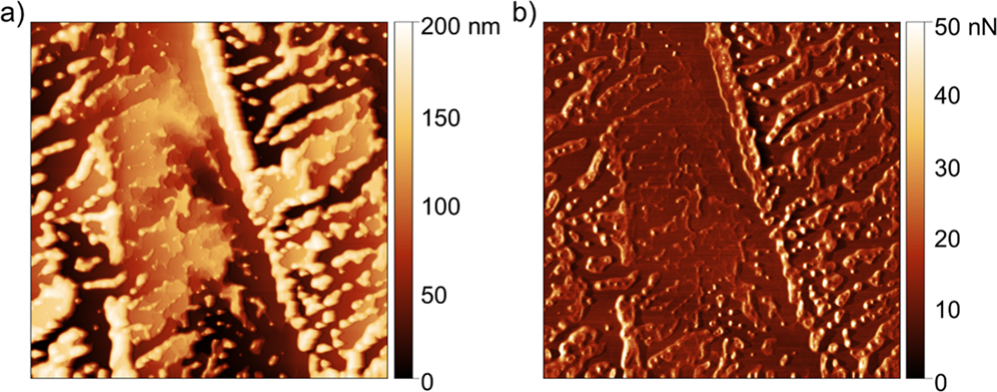
(a) Topography image and (b) corresponding friction force map on a freshly cleaved KCl (100) surface exposed for 6 h to BMIM-PF_6_. Frame sizes: 14 × 14 μm^2^.

To gather additional evidence on the presence of the adsorbate and gain insight into their composition, we have performed Raman spectroscopy on both NaCl and KCl after blowing off BMIM-PF_6_. The results are shown in Figure 6a–d. The KCl surface exposed to the IL has clearly visible bands at 742, 1025, 1064, 1128, and 1296 cm^–1^. Those peaks are related to BMIM-PF_6_ and can be assigned to ring or CCCC vibrations, according to Table 1.^23^ The 1064 cm^–1^ and 1128 cm^–1^ bands are characteristic fingerprints for the crystalline state.^24^ Furthermore, a close examination of the data presented by Saouane et al.^12^ reveals that these peaks are less intense in the γ-phase and have comparable intensity to the 1025 cm^–1^ band for the α-phase and β-phase, similarly to our case. The 1064 cm^–1^ and 1128 cm^–1^ bands are also observed for NaCl, although their intensity is much weaker than that for KCl (Figure 6c,d). Since the α-phase has an orthorhombic structure with a rectangular base of 9.39 × 9.78 Å^2^ deviating only slightly (4%) from a square, this phase is the most plausible in the present case. In addition, the mismatch with KCl (lattice constant *a* = 6.29 Å) is favorable (∼3:2), which is not the case with NaCl (*a* = 5.64 Å, i.e., ∼5:3 ratio). Under these conditions, the formation of a commensurate superstructure is more favorable on KCl.

**Figure 6:**
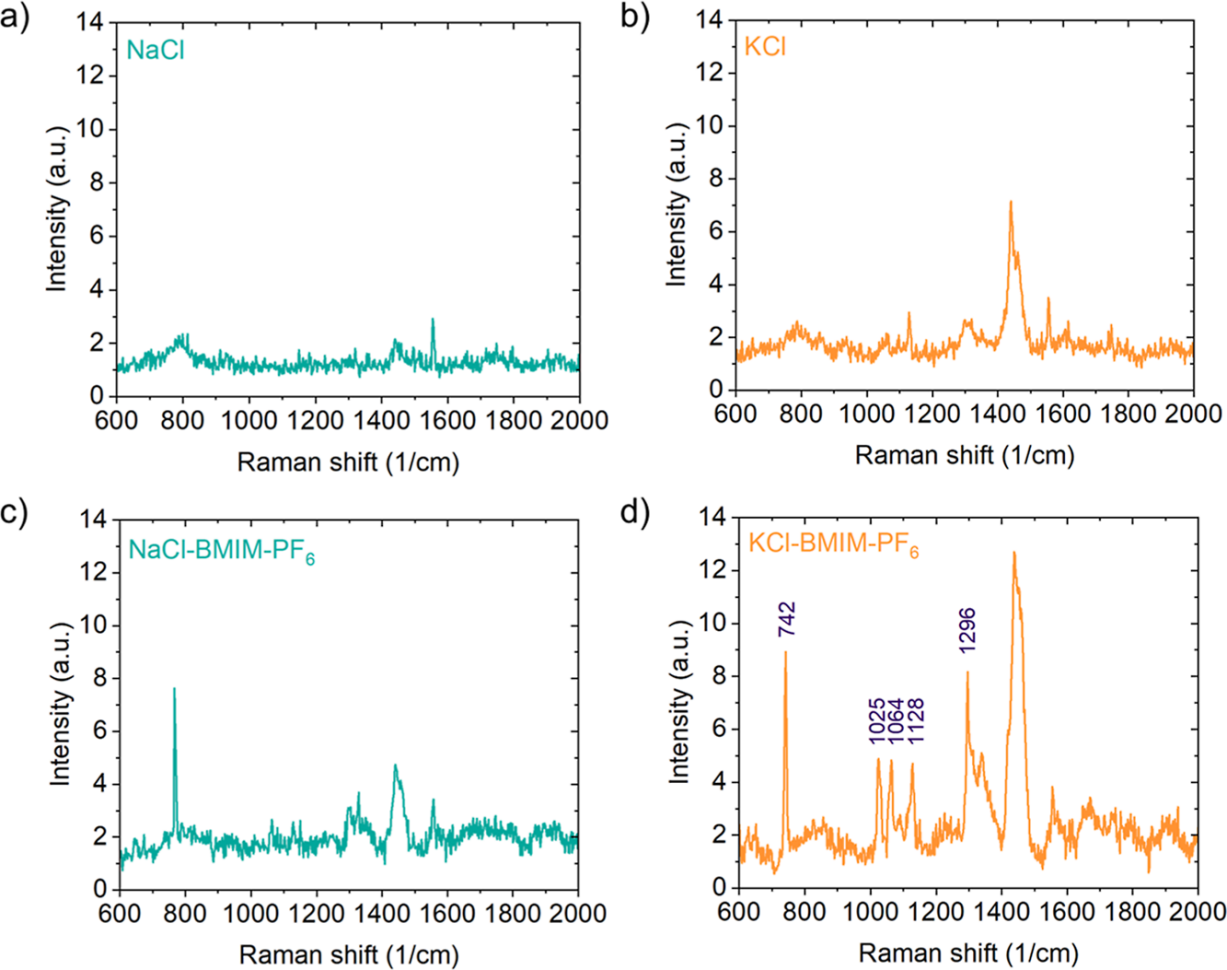
Raman spectra of (a) pure NaCl and (b) pure KCl surfaces as well as of (c) NaCl and (d) KCl surfaces after exposure to BMIM-PF_6_ and dry nitrogen blow-off of the wet IL layer.

**Table 1:** Raman Band Peaks of BMIM-PF_6_

Raman band [cm^–1^]	assignment
742	ring HCCH sym. bend and NC(H)N bend
1025	ring sym. stretching
1064	CCCC stretching
1128	CCCC stretching
1296	CCCC stretching, ring asym. stretching

To shed light on the different response of NaCl and KCl at the atomistic level, we have also performed MD simulations based on the configuration in Figure 1, with the results in Figure 7. Here, the charge density distributions in Figure 7a show a more pronounced peak of both [BMIM]^+^ cations and [PF_6_]^−^ anions near the KCl surface, with the maximum charge density occurring at *Z* = 2.83 nm for KCl, while *Z* = 3.65 nm for NaCl. This indicates a stronger interaction of the BMIM-PF_6_ IL with the KCl surface than with NaCl, suggesting a higher propensity for IL crystallization on KCl. Figure 7b presents the diffusion coefficients of the [BMIM]^+^ cation and [PF_6_]^−^ anion in the vicinity of NaCl and KCl surfaces (with all ions considered in the calculations). The results demonstrate that the diffusion coefficients of both cations and anions are significantly lower on the KCl surface than on the NaCl surface. In detail, the diffusion coefficients for [BMIM]^+^ and [PF_6_]^−^ are reduced by approximately 30% and 50%, respectively, when in contact with KCl. This reduction of ion mobility on KCl further confirms the stronger binding and reduced ionic motion near the KCl surface than NaCl, thereby supporting that BMIM-PF_6_ exhibits a greater tendency to form ordered structures (i.e., crystallites) on KCl. In line with these results, we should also remark that AFM imaging performed by Ichii et al. on KCl in the same IL using frequency modulation technique resulted in instabilities, which were attributed to possible dissolution of the substrate.^25^ On the contrary, a similar characterization on NaCl was stable.^26^ This is consistent with the abrupt dissolution that we observed on KCl (Figure 2d) but not on NaCl (Figure 2c). Finally, we estimated the electrostatic and van der Waals contributions separately for the two systems (Figure 7c). Interestingly, the electrostatic contribution is almost the same in both cases, whereas the vdW interaction is significantly larger for the KCl–BMIM-PF_6_ system.

**Figure 7:**
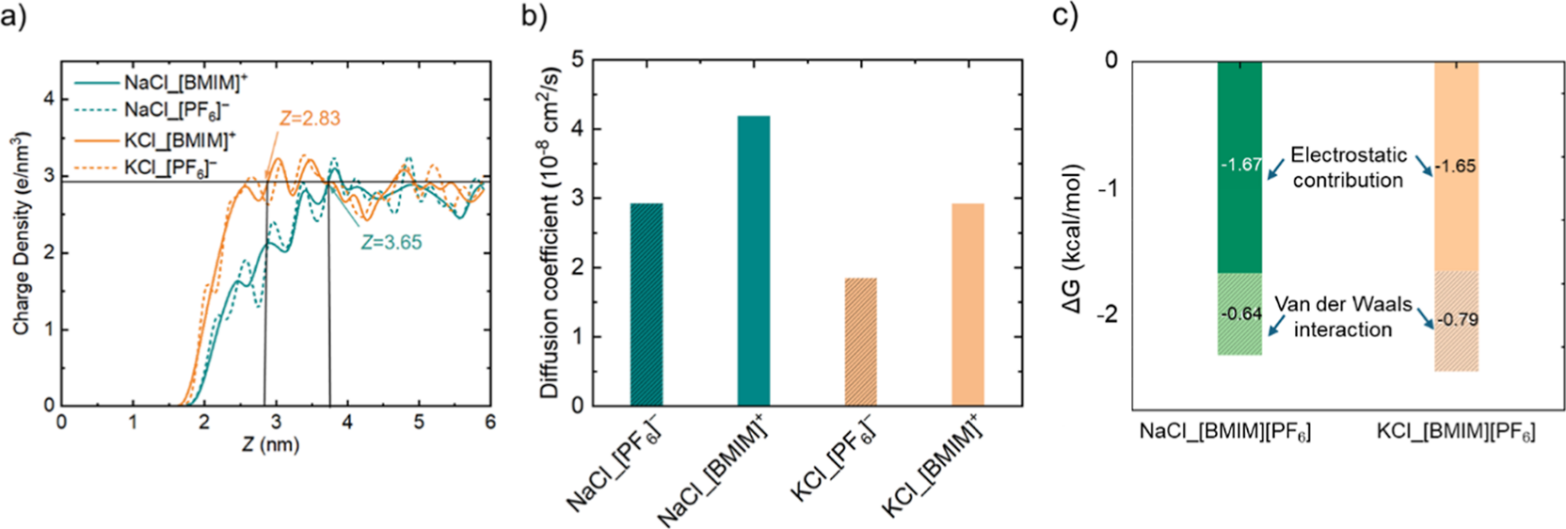
(a) Charge density profiles and (b) diffusion coefficient of [BMIM]^+^ and [PF_6_]^−^ at NaCl and KCl surfaces. *Z* indicates the distance of cations/anions to the NaCl and KCl surfaces. (c) Electrostatic and van der Waals contributions to the interaction energy between BMIM-PF_6_ and the NaCl and KCl surfaces.

## 4 Conclusions

To summarize, we have characterized the nanomorphological and -tribological response of NaCl and KCl (100) crystal surfaces exposed to the BMIM-PF_6_ IL for 6–7 h. The KCl surface is much more reactive than NaCl. Its top layers are dissolved immediately after contact with the IL. Afterward, the IL forms stable nanocrystalline islands on KCl, but only in the areas untouched by the AFM tip. On the other areas, the surface is simply smeared off. On the NaCl surface, there is no evidence of adsorbate formation. The surface is rather smoothed by the scanning tip with a considerable displacement of the step edges in the scan direction. Such a different behavior was never reported in the literature, to the best of our knowledge. Raman spectroscopy confirms the presence of crystalline structures on KCl compatible with the α and β solid phases of BMIM-PF_6_. MD simulations corroborate the experimental observations by attesting a much stronger attraction of the IL toward KCl. On the basis of these results, NaCl appears as a more suitable candidate than KCl for applications requiring long-term stability of an ionic crystal surface in the chosen IL.

## Supplementary Material







## Notes

Comparison of friction force maps of NaCl and KCl surfaces shortly after cleavage and after exposure to BMIM-PF_6_ (including prolonged scanning); selected images of crystallites formed on KCl exposed to BMIM-PF_6_ for different times; and time evolution of NaCl and KCl surfaces repeatedly scanned in BMIM-PF_6_ (PDF)
Structural change in NaCl steps during 6 h sliding (MP4)
Structural change in KCl steps during 7 h sliding (MP4)

## ○

Center for Advancing Electronics Dresden, TU Dresden, 01069 Dresden, Germany
