# Supplementary material for: Structural Stability of NaCl and KCl Cleavage Surfaces in the BMIM-PF6 Ionic Liquid
Source: Langmuir. 2025 May 30;41(22):13793–9. doi: 10.1021/acs.langmuir.5c00163 (PMC12164334; doi:10.1021/acs.langmuir.5c00163)
Supplement: Supplementary file 3 [file la5c00163_si_003.pdf]

Supporting Information:

Structural Stability of NaCl and KCl Cleavage Surfaces in BMIM-PF_6 Ionic Liquid

Ebru Cihan[1+], Natalia Janiszewska[2,3], Kamil Awsiuk[2], Qingwei Gao[4], Rong An[5,6], Ronen Berkovich[7,8], Enrico Gnecco[2]

[1]Institute for Materials Science and Max Bergmann Center for Biomaterials, TU Dresden, 01069 Dresden, Germany

[2]Marian Smoluchowski Institute of Physics, Faculty of Physics, Astronomy and Applied Computer Science, Jagiellonian University, 30348 Krakow, Poland

[3]National Synchrotron Radiation Centre SOLARIS, Jagiellonian University, Czerwone Maki 98, PL-30392 Krakow, Poland

[4]College of Environmental and Chemical Engineering, Shanghai Key Laboratory of Materials Protection and Advanced Materials in Electric Power, Shanghai University of Electric Power, Shanghai 200090, China

[5]School of Materials Science and Engineering / Herbert Gleiter Institute of Nanoscience, Nanjing University of Science and Technology, Nanjing 210094, China

[6]Shandong Laboratory of Advanced Materials and Green Manufacturing at Yantai, Yantai 264006, P.R. China

[7]Department of Chemical Engineering, Ben-Gurion University of the Negev, Beer-Sheva, 8410501, Israel.

[8]Ilze Katz Institute for Nanoscience and Technology, Ben-Gurion University of the Negev, Beer-Sheva, 8410501, Israel

[+]Current address: Center for Advancing Electronics Dresden (cfaed), TU Dresden, 01069 Dresden, Germany

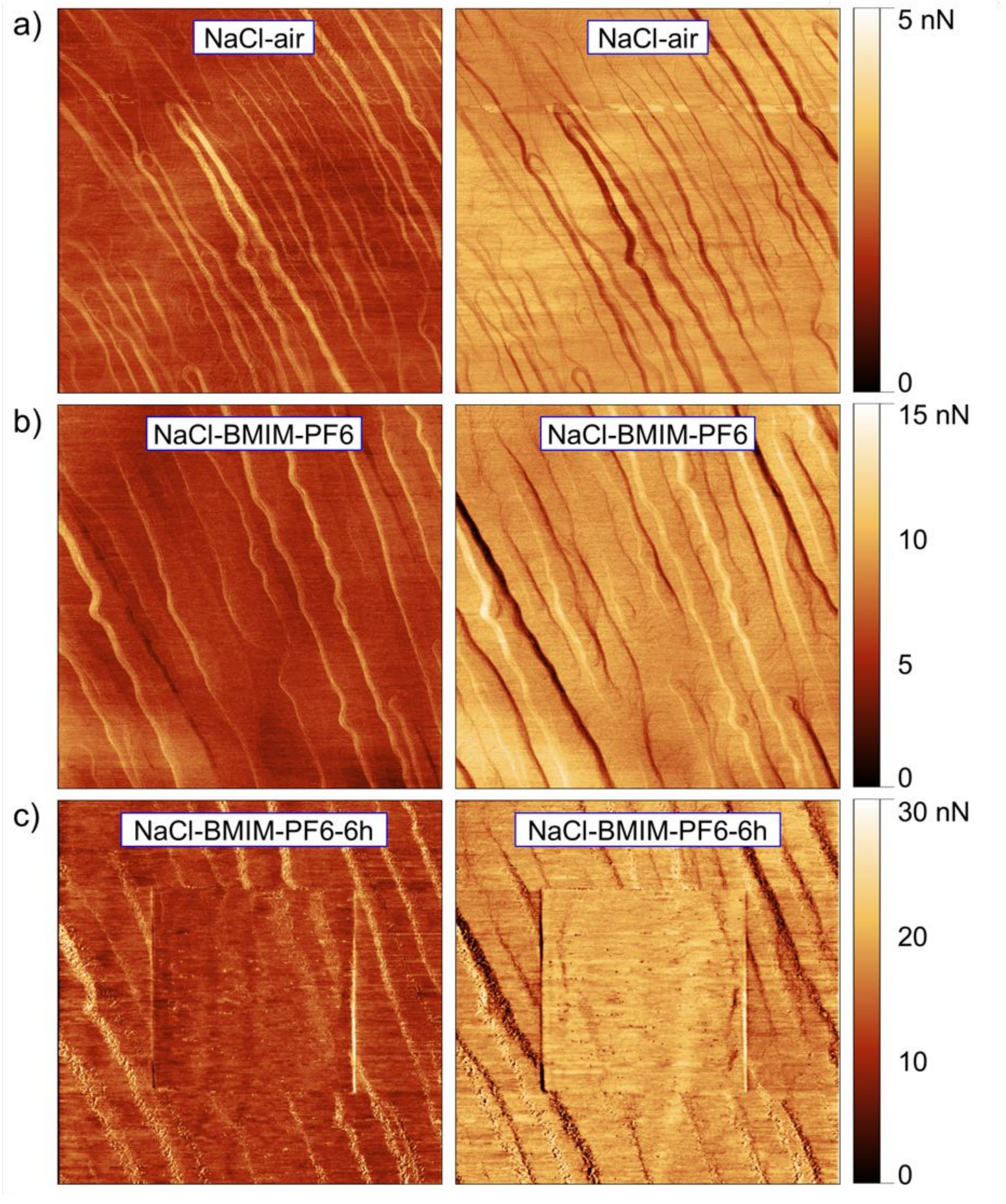

Figure S1. AFM friction force maps of NaCl surface measured **(a)** in air, **(b)** in BMIM-PF_6 ionic liquid and **(c)** in BMIM-PF_6 after 6 h. The images on the left are friction force maps for the forward scan and the images on the right are friction force maps for the backward scan. Frame sizes: 14×14 μm^2. Normal force: F_N < 10 nN.

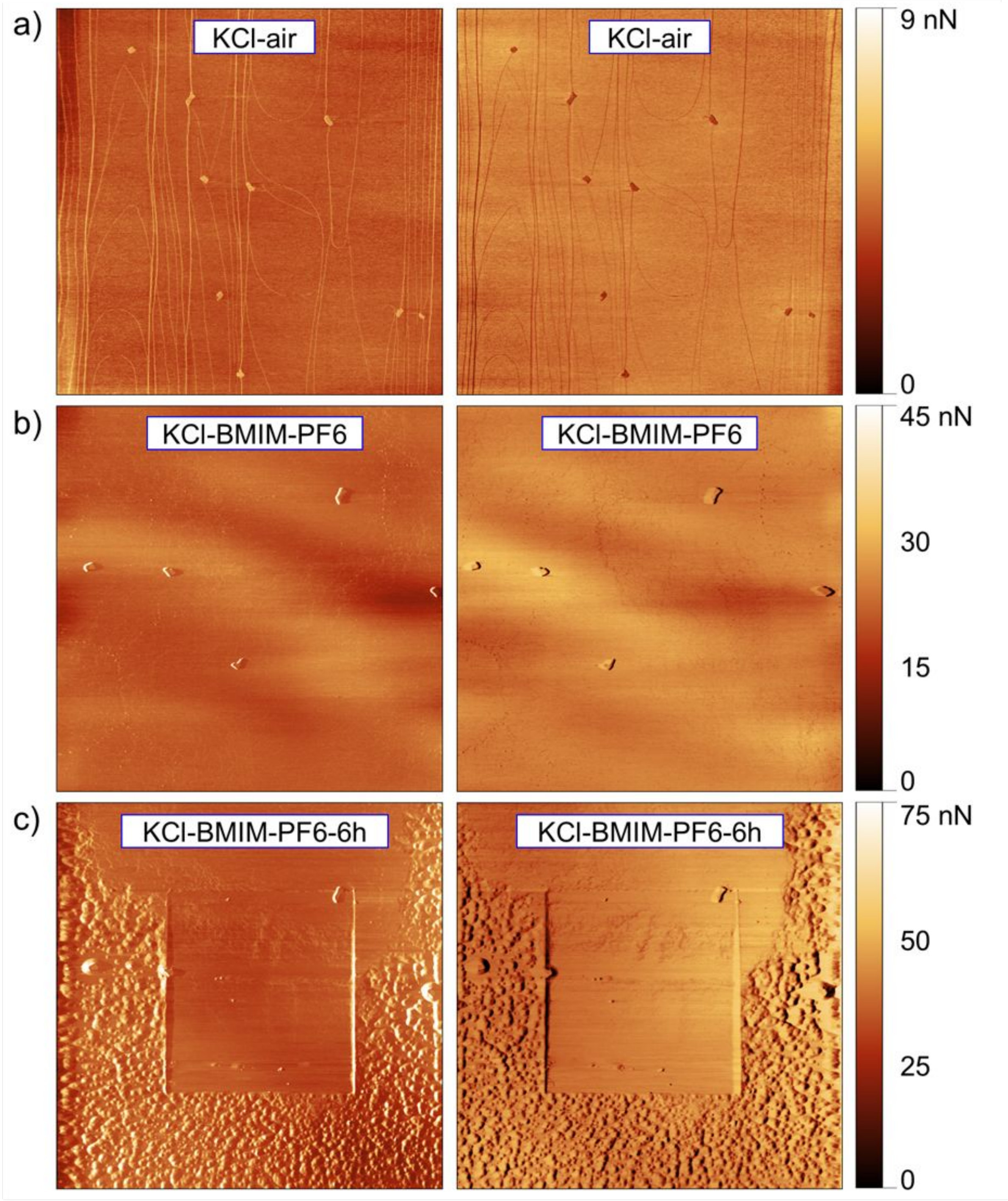

Figure S2. AFM friction force maps of KCl surface measured **(a)** in air, **(b)** in BMIM-PF_6 ionic liquid and **(c)** in BMIM-PF_6 after 7 h. The images on the left are friction force maps for the forward scan and the images on the right are friction force maps for the backward scan. Frame sizes: 14×14 μm^2. Normal force: F_N < 10 nN.

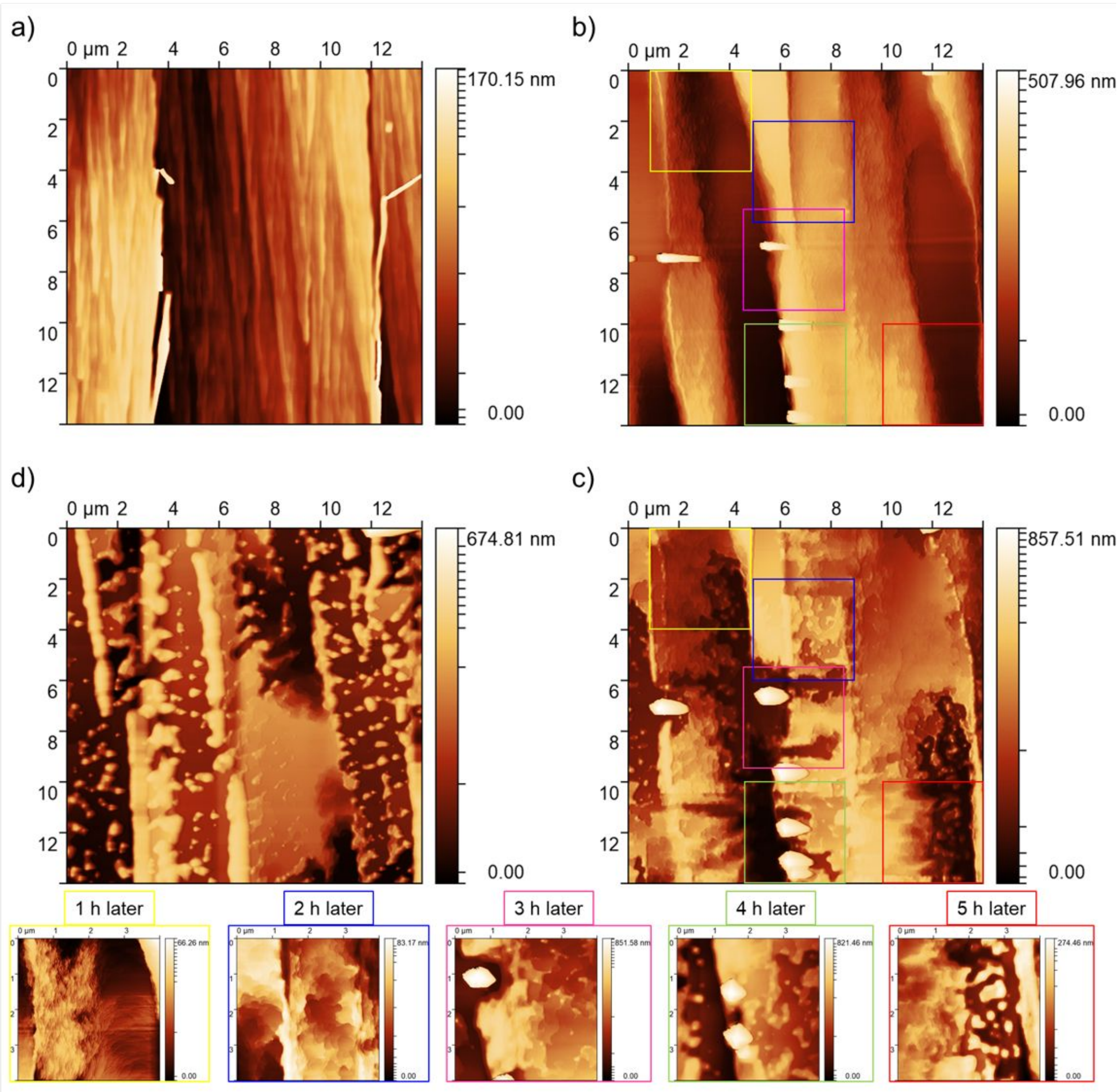

Figure S3. High-step AFM topography images of the freshly-cleaved KCl surface measured **(a)** in air and **(b-c)** in BMIM-PF_6 ionic liquid. The image in **(b)** was taken within the first half hour of exposure to the IL. Smaller areas, indicated by squares of different colors, were then scanned every hour, focusing on one of them at a time (shown as thumbnails below). The image in **(c)** was taken 6 h later in the same area, only the small squares were scanned once and areas outside these squares were not scanned. The scanned areas showed abrasion due to the duration of exposure to the ionic liquid rather than crystallite formation. Also note that small crystals already present on the surface form larger crystals when exposed to the ionic liquid. **(d)** AFM topography image of another area exposed to the ionic liquid at the end of 6 h and not scanned during this 6 h period. The ionic liquid molecules have coalesced at the steps on the KCl surface, forming adlayers. Normal force: $F_N < 10$ nN.

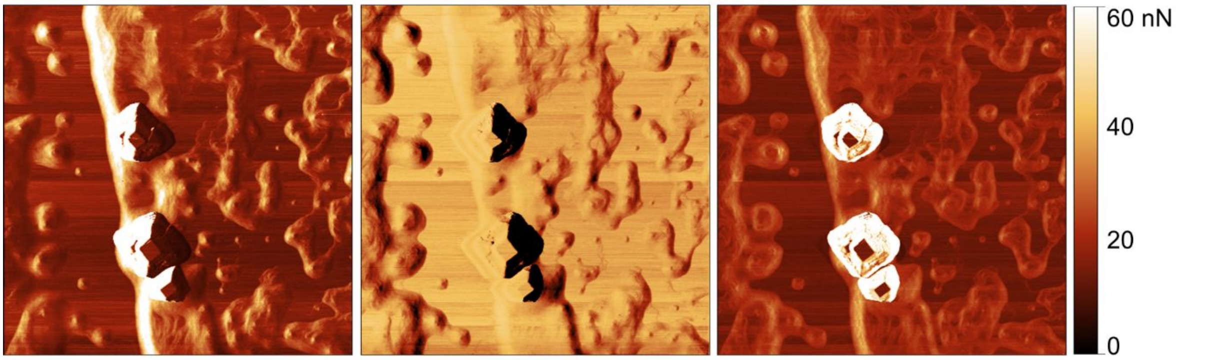

Figure S4. Friction force map of crystals growing after 4 h on the KCl surface, shown in Figure S3b-c. It can be observed that the friction force on the crystals is greater than on the surface. The map on the left depicts the friction force in the forward direction, while the map in the middle represents the friction force in the backward direction. The map on the right is the result of subtracting the friction forces in the forward and backward directions. Frame sizes: 4×4 μm^2. Normal force: $F_N < 10$ nN.
